# 

**Published:** 1810-12

**Authors:** 


### MONTHLY CATALOGUE OF NEW MEDICAL PUBLICATIONS.

---

Remarks on the Nomenclature of the New London Pharmacopœia, read before the Liverpool Medical Society. By John Bostock, M. D. 8vo. Callow.

Observations on the Cure of Cancer. By Thomas Denman, M. D. 8vo. Johnson.

An Enquiry into the Causes producing the extraordinary Addition to the Number of Insane, together with extended Observations on the Cure of Insanity; with Hints as to the better Management of Public Asylums for Insane Persons. By W. S. Hallaran. 8vo. Longman and Co.

On the Morbid Sensibility of the Eye, commonly called Weakness of Sight. By John Stevenson. 8vo. Highley.

A Popular Essay on the Structure, Formation, and Management of the Teeth; Illustrated by Engravings. By John Fuller. 8vo. Sherwood and Co.

Facts and Opinions concerning Diabetes. By John Latham, M. D. 8vo. Murray.

The Elements of Chemistry. By J. Murray. 2 vols. 8vo. Underwood.

The London Medical Review, No. 12. No. 13 will be published in January. Underwood.

Pharmacopœia Officinalis Britannica, being a new and correct Translation of the late Edition of the London Pharmacopœia, with which are incorporated, in Alphabetical order, all the Formulæ of the Edinburgh and Dublin Colleges; together with Notes Explanatory of the different Processes. By Richard Stocker. 8vo. Cox.

Illustrations of Madness: exhibiting a singular case of Insanity, and a no less remarkable Difference in Medical Opinion; developing the Nature of Assailment, and Manner of Working Events; with a Description of the Tortures experienced by Bomb-bursting, Lobster-cracking, and Lengthening the Brain. Embellished with a curious plate. By John Haslam, 8vo. Rivington's.

The Elements of Experimental Chemistry. By William Henry, M. D. F. R. S. 2 vols. 8vo. Johnson.

First Lines of the Practice of Physic. By William Cullen, M. D. including the Definitions of the Nosology, with Supplementary Notes. By Peter Reed, M. D. 2 vols. 8vo. Underwood.

### NOTICES TO CORRESPONDENTS.

We are much obliged to our old and valuable correspondent Mr. Goodwin for his interesting communication. We had heard of the ravages of Scarlatina Maligna in various districts, but were not aware, that they had been so extensively fatal as our correspondent narrated. We do not presume to dictate any mode of treatment, but as that which Mr. Goodwin pursued, and which indeed has been recommended bythe best practical writers on the subject, in many instances failed, and in none seemed to be beneficial, we suggest the propriety of directly reversing it, of strictly enforcing what is termed the antiphlogistic plan; and more especially in the commencement of the disorder, administering purgatives, and applying the cold affusion. On this important and interesting snbject, we earnestly solicit communications from practitioners who have tried the effect of this practice in the more severe forms of the disease. We also wish them to direct their attention to any local circumstances which might influence the fatality of a complaint, which in many instances is extremely mild, and in others again, running its course with rapidity, assuming the most malignant character, and resisting the effect of remedies.

We are obliged to "*Socrates*" for his hints, and when he is better acquainted with us, hope that he will not dispute our intention of performing our duty to the public with fidelity.—Upon referring to the Gentleman's Magazine for September 1762, we could not find the case of Premature Puberty to which he alluded.

We have to acknowledge communications from Dr. Arnold, Dr. Hamilton, Dr. Clough, Mr. Goodwin, Mr. Kenworthy, and Mr. Chamberlain, which will appear in our next number.

---

END OF VOL. XXIV.

*Printed by E. Hemsted, Great New Street, Fetter Lane.*

### INDEX.

Abdomen, a case of tumour in the 105
Abernethy, Mr. on cancer 307
Abortion, on a trial for procuring 38
Abscesses, on the treatment of 84
Abstinence, on a supposed case of 309
Acid, effects of magnesia on uric 203
Acids, on the combination of 321
Action, cases of translation of diseased 178
Advertisement to the readers of the Medical Journal 349
Adams, Dr. on epidemics 22
——— Mr. on the Eau Medicinale D'Husson 364
Alcohol, on the efficacy of 98
Alderson, Dr. on apparitions 161
Aloe, description of the varieties of 528
Agrimony, description of 517
American army, mortality in the 435
Aneurism, on a case of venous 285
Animal bodies, on the effluvia of dead 422
Animals, on the torpidity of 8
Animalcula, diseases generated by 23
Anus, case of imperforate 518
Anus, on diseases of the 415
Apoplexy, case of fatal 92
Apparitions, on the belief of 161
Arnell's, Dr. case of supposed poison 373
Arsenic, on the method of detecting 59
——— on an additional test for 145, 274
——— on preparations of 403
Arteries, on the functions of the 9
Artery, effect of a ligature on the iliac 524
Ascites, case of 140
Asthmatic fits occasioned by ipecacuanha 61, 233
Aurochs, discovery of an animal of the race of 87
Bacot, Mr. on the belladonna 383
Baird, Dr. on the ship ulcer 13
Barton, Dr. on the torpidity of swallows 187
Bath, description of an improved vapour 34
——— on constructing a steam 162
Bell, Mr. on professional character 343
Belladonna, singular effect of 383
Beer, Dr. on epidemical ophthalmia 317
Bile, observation on the use of the 237
——— for medical improvement 85, 173
Bladder, case of diseased 463
Blistering fly, account of a new, 430
Blood, case of extravasation of 89
Blood-letting in palsy, on 375
Bohan upas, or poison tree, account of the 174
Books, on the multiplication of 2
Books, critical analysis of medical.——Thomæ Simsoni, Dissertationis, Quatuor, &c. 64. A practical Essay on Cancer, by C. T. Johnson, 72. A Conspectus of the Pharmacopœias of London, Edinburgh and Dublin. By A. Todd Thompson, 80. An Essay on the Nature of Scrofula. By Richard Carmichael, 148. The Edinburgh Journal, No. xxiii, 157. Letter in reply to the Report of the Surgeons of the Vaccine Institution, Edinburgh. By T. Brown, 163. A memoir on the physiology of the Egg. By J. A. Paris, M. D. 164. Practical Observations on Disorders of the Stomach. By G. Rees, M. D. 257. Analysis of a treatise on the plica polonica. By F. de la Fontaine, 329. A Conspectus of the London, Edinburgh, and Dublin Pharmacopœias. By R. Graves, M. D. 340. An account of Spina Bifida. By T. V. Okes, 341. Letters on Professional Character and Manners. By J. Bell, 343. A Dissertation on Retroversion of the Womb. By S. Merriman, M.D. 406. Observations on Diseases of the Rectum and Anus. By T. Copeland, 415. The Edinburgh Medical and Surgical Journal, No. xxiv. 422. Hortus Kewensis. By W. Aiton, 428. Geoghegan on the treatment of ruptures, 512. Address to the Lincolnshire Medical Society, by E. Harrison, M. D. 494. Description of an Affection on the Tibia, by T. Whately, 501. On Ruptures, by E. Geoghegan, 512
Botanical report, monthly 438, 528

Bottles, method of preparing phosphorus 86
Bradley, Dr. on strangulated hernia 112, 193
——— on a case of venous aneurism 285
Brain, observations on diseases of the 52
——— case of diseased 89
Brande, Mr. on magnesia 203
Bree, Dr. remarks on 357
Brierley's, B. case of 463
Brodie's, Mr. description of a fœtus 5
Brown, Mr. on vaccination 163
Burns, Mr. on the heart 27
Burns, and scalds, efficacy on alcohol in 98
Bush's, Mr. case of obstruction in the urethra 147
Cadet, M. on the unwholesomeness of tea 189
——— remarks in reply to 265
Calculus taken from the bladder after death 185
Calomel in dysentery, on the use of 161
Calomel in fever, on 461
Camelopardus giraffa, skeleton of the 432
Cancer, practical essay on 72
——— cases of 275
——— Dr. Lambe's report on 306
Cantharides, efficacy of in fluor albus 277
Carditis, case of 426
Carmichael, Mr. on scrofula 148
Cases—Of hydrophobia, 29. Of tetanus, 40. Of syphilis cured by opium, 45. Of diseases of the brain, 52, 89. Asthma, 61. Monstrosity, *ibid.* Secondary small-pox, 86. Of fatal apoplexy, 92. Of tumour in the abdomen, 105. Of puerperal fever, 107. Strangulated hernia, 112. Egyptian ophthalmia, 130. Tumour of the tongue, 134. Of ascites, 140. On Mr. Windham's, 142. Obstruction in the urethra, 147. Of scrofula, 150. Of diabetes, 157. Of herpes, 162. Of translated diseased action, 178. Of metastasis, 181. Of translated morbid action, 183. Of calculous complaints, 185, 204. The itch, 214. Herpes, 220. Of superfœtation, 232. Of asthmatic fits, 233. Salivation, 241. Dropsy, 242. Of flatulence, 248. Of a tumour, 261. Of gravel, 267. Of a foreign body in the trachea, 269. Of cancer, 275. Fluor Albus, 277. Of hæmatocele, 285. Fungus hæmatodes, 289. Hæmorrhage in amputation, 299. Plica polonica, 337. Of ulcerated tongue, 356. Suppression of urine, 360. Of violent operation of the Eau Medicinale D'Husson, 364. Of marsh fever in America, 365. Supposed poison, 373. Of the efficacy of bleeding and cathartics in palsy, 377. Of contusion, 381. Effect of belladonna in nervous affection, 383. Of premature puberty, 390. Of the bite of a rattle-snake, 391. Of retroversion of the womb, 409, 412. Of an uncommon disease of the throat, 425. Of carditis, 426. Of secondary small-pox, 430. Of the effects of lightning, 437. Of diseased bladder, 463. Scarlatina anginosa maligna, 465. Of dislocated shoulder, 468. Of diseased Tibia, 502. Of chorea, 476. Of croup, 478. Of tænia, 517. Hydrothorax, *ibid.* Worms, 518. Imperforate anus, *ib.*
Catarrh, on the treatment of 253
Cathartics, their efficacy in palsy 375
Charcoal, on combustion of 347
Chesnut, use of the horse 430
Chisholm, Dr. on the effluvia of dead bodies 422
Chorea, observations on 476
Cinchona, on its use in ophthalmia 221
Clarke's, Dr. medical report 157
Clarke, Dr. on calomel 461
Clusius, account of 449
Cobra di capello, on the bite of the 400
Cochrane's, Mr. vapour bath described 34
Cold, its effects on the human body 69
——— mode of producing artificial 347
Cole, Dr. on marsh fever 365
College of physicians, annual meeting of the 431
Contagion ascribed to animalcula 25
Contusion followed by retention of urine 381
Copeland, Mr. on diseases of the rectum and anus 415
Correspondents, to 88, 264, 443, 531
Cranium, description of an uncommon 87
Crawford, Dr. on yellow fever 23
Croup, treatment of the 254, 478
Cupping, on the practice of 177
Cureaudeau, M. on an artificial stony substance 316
Currie, Dr. an observation of 1
Cutaneous affections, treatment of 255
Cynanche tonsillaris, on the treatment of 253
Croonian lecture, the 485
Croup, observations on 478

Cow-Pock Institution at Dublin, report of 518
Davy, Mr. honourable testimony of the French chemists to 346
Didier, Ant. character of 25
Defournelle, Dr. remarkable age of 431
Dessault, his notion of syphilis 25
Diabetes, case of 157
Diarrhœa, observations on 254
Digestion, use of the bile in 237
Diseases, antient mode of curing 67
——— on the danger of curing certain 121, 214
——— on the cause of inflammatory 230
——— of the stomach, observations on 237
——— of Edinburgh, reports of 83, 172, 252
——— in London, reports of 81, 171, 255, 347, 429, 516
Dispensaries of heat, on 358
Dislocations, on reducing 468
Dogs, on the distemper of 31
Dreams, observations on 284
Dropsy, treatment of 85
Dublin, report of the Cow-Pock Institution at 518
—— Foundling Hospital 522
Dryander, Mr. death and character of 441
Dysentery, account of an epidemic 161
Earle's, Sir James, account of a calculus 185
Earthquake at the Cape of Good Hope 87
Eau Medicinale d'Husson, observations on the 351, 474
——— violent operation of 364
——— anti-arthritique, analysis of the 431
Edinburgh, report of the diseases of 83, 172, 252
——— Medical and Surgical Journal, review of 157, 422
Education, on medical 303
Effluvia, on putrescent 422
Egg, on the physiology of the 164, 237
Electric column, description of an 86
Emetics in horses, effects of 271
Epidemical Ophthalmia, on 317
Epidemics, observations on 22
Extravasation of the blood, case of 89
Empyema, on the 517
Eupatorium cannabinum, on the 517
Experiments.—On arsenic, 59, 145; on the bohan upes, or poison tree, 175; on teas, 190, 266; on the composition of the urine, 207; on potash, 227; on animal corruptible substances, 321; on the muriatic acid, 345; on charcoal, 347; on the Eau de Pradier, 430.
Fever, on the yellow 23
—— case of puerperal 107
—— cases of marsh 365
—— on Dr. Hamilton's method of curing 430
—— on calomel in 461
Finsbury dispensary, state of inoculation at the 148
Fistula in ano, on the 421
Fits, case of violent asthmatic 233
Flatulence, case of 248
Fluor albus, case of 277
Fly, account of a new blistering 430
Fœtus, account of a remarkable 5, 7
Forbes, Mr. on the steam bath 162
Foster's, Mr. meteorological diary 176
Freer, Dr. strange hypothesis of 24
Fungus hæmatodes, on the 11
Foundling Hospital, Dublin, report of the 522
Gall, Dr. on the system of 3
Gastritis, treatment of 162
Gestation, on 491
Good Hope, earthquake at the Cape of 87
——— epidemic dysentery at 161
Goodwin, Mr. on Scarlatina 465
Gravel, on a case of 267
Graves's, Dr. conspectus of pharmacopœias 340
Gregory, Dr. on sleep 279
——— on the conduct of 343
Grenville's Dr. case of herpes 162
Hall's, Dr. case of tumour on the tongue 134
—— remarks on odours 223
Hœmorrhoidal excrescence, on the 420
Hamilton's, Dr. case of puerperal fever 107
——— case of gravel 267
——— Mr. on the use of cinchona, 221
Hargrove, Mr. on emetics 271
Harrison's, Dr. case of syphilis 45
——— memorial on medical education 303
——— Mr. cases of cancer and fluor albus 275
——— Dr. address 494
Headley, Mr. on ophthalmia 130
—— reply to 221
Heart, on the functions of the 9
—— Mr. Burn's on the 27
Heat, on the dispensaries of 358
Helicis, on the British 19
Hernia, an alleged remedy for 18
———, observations on strangulated 112, 193

Herpes exedens vermiculatus, case of, 862
—— observations on 219
Hill's, Mr. case of monstrosity 61
—— observations on Mr. Windham's case 142
Historical sketch of the progress of medicine 1
Home's Mr. account of the squalus maximus 5
—— case of the bite of a rattle-snake, 391
Horses, on the effect of emetics in 271
Hortus Kewensis, account of the 428
Hottentot, description of a female 385
Houzouanas, description of the 388
Howorth, on the case of Philip 389
Howship, Mr. on diseases of the brain 52, 89, 92, 178 89, 181, 183
Hume's Mr. test for the detection of arsenic 145, 403
Hydrophobia, case of 29
—— remarks on 46
Hydrocephalus internus, on 210
Hysteralgia, observations, on 81
Hydrothorax, case of 517
Index, on a general 308
Inflammatory diseases, cause of 230
Inoculation, statements of vaccine 86, 148, 174
Inquirer, the medical 162
Ipecacuanha, on the peculiar property of 60, 223, 233
Iron, efficacy of carbonate of 134
Itch, observations on the 125
Iliac artery, effect of a ligature on the 524
Jacksonian prize subject for 1811 261
James I. his opinions on tobacco 447 456
James, Dr. anecdote of 475
Jenner, Dr. on the distemper in dogs 31
Johnson, Mr. on cancer 72
Jones's, Mr. case of tetanus 40
—— on the detection of arsenic 59, 274
Jenner, Dr. on vaccination 520
Kentish, Dr. on dispensaries of heat 358
Kenworthy's, Mr. case of diseased bladder 463
Kinglake's, Dr. case of tumour 105
—— cases of a foreign body in the trachea 269
Kircher, character of 24
Knight, Mr. on the Alpine strawberry 87
Knowles's, Mr. case of ascitis 140
—— use of nitrous acid 355
La Grange's, Mr. report on pharmacy 224
Lambe's, Dr. report on cancer 306
—— use of raw vegetables 363
Langius, account and character of 24
Lectures announced.—Dr. Clough on Midwifery, 88, 263. Dr. Squire on the same, *ibid.* at Glasgow university, 262. St. Thomas' and Guy's hospitals, *ibid.* Dr. Benton on the Practice of Medicine, *ib.* Mr. Carlisle on Surgery, *ib.* M. Carpue on Anatomy, &c. 263. Dr. Clarke on Midwifery, *ib.* Dr. Clutterbuck on the Theory and Practice of Physic, &c. *ib.* Dr. Dennison on Midwifery, *ib.* Drs. Hooper and Ager on Physic, &c. *ib.* Mr. Moor on the Teeth, *ib.* Dr. Ramsbotham on Midwifery, 264. Dr. Reid on the Theory and Practice of Medicine, *ib.* Dr. Roberton, on Midwifery, *ib.* Mr. Shute on Anatomy, &c. *ib.* Dr. Tuthill on the Practice of Physic, *ib.* At the Theatre of Anatomy, Great Windmill-Street, 344. Mr. Chevalier on Surgery, 345. Dr. Squire on Midwifery, *ib.* Dr. Pearson's on Medicine, *ib.* Dr. Farre and Mr. Traver's on the changes produced by disease in the body. *ib.* Dr. Adams on Medicine, 346. Mr. Brooks on Anatomy, &c. 431. Mr. Macartney on Comparative Anatomy, 432. At the Middlesex Hospital, *ib.* Mr. Singer on Electricity, *ib.* Mr. Armiger on Anatomy, *ib.* Dr. Clough's, on Midwifery 526
Le Fevre, Dr. account of 351
Leslie, Mr. on artificial cold 347
Lichtenstein, Dr. on an epidemic dysentery 161
Ligature on the iliac artery 524
Lightning, curious effects of 437
Lincolnshire benevolent society, resolutions of the 173
Linnæus on the animalcular hypothesis 26
London, reports of diseases in an eastern district of 81, 171, 255, 347, 429, 516
—— vaccine institution, report of the 174
Longevity, remarkable instance of 431
—— of a tortoise 525
Magnesia, on the effects of 203
Mantisia saltatoria, description of 530
Maryan, Mr. on the hydrophobia 46
Medical science, account of the bill for improving 85, 173
—— education, memorial on 303
Medicine, sketch of the progress of 1
Merriman, Dr. on suppression of the urine 360
——, on retroversion of the womb 406
Metastasis, case of 181

Meteorological diary 176
————, table 444
Medical and Physical Intelligence 85, 173, 261, 344, 430, 517
Medical men, society for the relief of their widows 471
Meteorological table 531
Monstrosity, case of 61
Moor, Ann, on the case of 309
Morbid action, case of, translated 183
Mottles, Dr. on the wholesomeness of tea 265
Muscles surrounding the urethra, on the 6
Musk, on the original use of 236
Mutis, Dr. memoir of 434
Muscular action, on 485
Middlesex hospital, benefactions to 526
Naval practitioners, on 163
Neck, case of fatal tumour of the 261
Nervous affection, effects of belladonna in 383
Nitrous acid, its use in ulcerations 356
Nottingham, medical report for 157
Nicot, Jean, account of 448
Nottingham vaccine institution, report of the 523
Oakes, Mr. on spina bifida 341
Odours, on peculiar effects from certain 223
Ophthalmia, use of cinchona in 221
————, cases of Egyptian 130
————, queries on epidemic 317
Opium, its use in syphilis 45
Orleans, sickness in the army at New 435
Palsy, efficacy of bleeding and cathartics in 375
Paris, report to the society of pharmacy at 224
Paris, Dr. on the physiology of the egg 164, 237
Parkinson, Dr. on alcohol in burns 98
Parturition, suppression of urine after 360
Pettigrew, Mr. on the brain 5
Peiresc, anecdote of 454
Pharmacopœias, on the London, Edinburgh and Dublin 80, 340
Phenomenon, account of a remarkable 435
Phosphorus bottles, method of preparing 86
Physic, intended translation of the fathers of 438
Physician, on the duties and qualifications of a 343
Physicians, meeting of the College of 431
Pigeons, remark on 169
Plane trees, on the destruction of 433
Plecis, Dr. opinion of contagion 25
Plica polonica, on the 329
Poison tree of Java, account of the use of 174
———— a disease supposed to arise from 373
Potash, on preparing the acetate of 224
Professional character, letters on 343
Puberty, on premature 389
Publications, lists of new medical 175, 432, 442
Puerperal fever, case of 107
Putrefaction, observations on 423
Quack, account of a remarkable 351
Quickening, observations on 38
Rattle-snake, case of a man bitten by a 391
Raymond, M. on the danger of curing certain diseases 121, 214
Rectum, on diseases of the 415
Rees, Dr. on disorders of the stomach 257
———— observations on 357
Reeve, Dr. on the torpidity of animals 8
Respiration, on disordered 357
Retroversion of the womb, on 407
Richerande, M. sketch of surgery 16
Roberton's, Dr. reports of diseases 33, 172, 252
———— observations on hydrocephalus internus 210
———— remarks on the opinions of 313
Rolfe's, Mr. case of superfetation 232
Royston's, Mr. sketch of the progress of medicine 1
———— reply to Dr. Lambe 465
Riding, salutary effects of 491
Salisbury, Mr. on the plane tree 334
Scalds, efficacy of alcohol in 98
Scarlatina anginosa, on the 465
Scarificator, description of a new 177
Schenk, Dr. on croup 478
Scott, Dr. on ipecacuanha 233
Scrofula, on the nature of 148
Sherril, Dr. on palsy 375
Ship ulcer, on the 13
Shute's, Mr. description of a new scarificator 177
Simpson's, Prof. four dissertations 64
Skinner, Mr. on a general index 308
Sleep, observations on 279
Smith's, Dr. case of contusion 381
Smallpox, causes of secondary 86, 430
Smut in wheat, on the 37
Snails, their use in hernia 18
—— observations on 20
Snakes, on the poison of 401
Snow, account of red 435
Spina bifida, account of 341

Squalus maximus, dissection of the 5
Stancliffe, Dr. on sleep 279
Steele, Mr. on the torpidity of swallows 187
Stomach, on disorders of the 257
Stony substance, on an artificial 316
Strawberry, mode of cultivating the alpine 87
Suffocation, case of death by 178
Superfetation, on the doctrine of 127
——— case of 232
Surgeons, on the education of 343
——— observations on sea 163
Swallows, on the torpidity of 187, 256
Sweats, on habitual 122
Sylvester, Joshua, on tobacco 354, 443
Syphilis attributed to animalcula 25
——— a case of 45
Scarlatina anginosa maligna, cases of 465
Sea sickness, on 488
Society for the relief of widows, &c. 471
Tarenne, M. on hernia 18
Taunton, Mr. on inoculation 148
Tea, on the properties of 189, 265
Tetanus, case of 40
Thenaud, M. on the combination of acids 321
Thermometer, new scale for the 191
Thomson's, Mr. conspectus of pharmacopœias 80
——— observations on a supposed case of abstinence 309
Throat, remarkable disease of the 425
Toad, curious circumstance concerning a 87
Tobacco, curious account of 354, 445
Tongue, case of tumour on the 134
———, on nitrous acid in ulceration of the 355
Torpidity of animals, on the 8
Trachea, effects of foreign bodies in the 269
Trotter, Dr. on ipecacuanha 60
Tumour in the abdomen, case of 105
——— tongue, case of 134
——— neck, case of 261
Thorius, R. account of 453
Tibia, on a disease of the 501
Tobacco, on the history and use of 445
Testudo Græca, longevity of 525
Tænia, cases of 517
Tortoise, longevity of a 525
Ulcer, on the ship 13
Ulcers, on the treatment of 84
Ulcerations, use of carbonate of iron in 134
——— of the tongue, efficacy of nitrous acid in 355
Upas tree, account of the 174
Urethra, on the muscles of the 6
——— dilatation of the male 524
——— case of obstruction in the 147
——— on stricture of the 313
Uric acid, effects of magnesia on the 203
Urine, on suppression of 360
———, remarkable case of 381
Vaccination, remarks on 32
Vaccinating, mode of 479
Vaccine establishment, statement of inoculation at the stations of 86
——— reply to the Edinburgh report on 163
——— annual report of the London 174
Variety of the human species, case of 385
Vegetable diet, on a 307, 363
Vegetable substances, on the combination of acids with 321
Venous aneurism, case of 285
Vesicle, description of the vaccine 479
Walcheren epidemic, on the 22
Walker's, Mr. new scale for the thermometer 191
Wheat, on the smut of 37
Williamson, Mr. on stricture in the urethra, 313
Windham, Mr. on the case of 142
Wilson, Mr. on the muscles of the urethra 3
Womb, on retroversion of the 406
Worms, case of 518
Whateley, Mr. on a disease of the tibia 501
Widows, account of a society for the relief of 471
Wilmer, Mr. on dislocations 468
Woollaston's, Dr. Croonian lecture 485
Yellow fever, on the 23
Young's, Mr. case of fœtus 9
——— Dr. Croonian lecture 6

### REFERENCES TO THE PLATES.

Mr. Howship's case of diseased Brain, plate I. - page 57
———, plate II. - - - 89
Mr. Shute's Scarificator - - - - - - - 177
Female from the interior of Africa - - - - - - 385
Mr. Kenworthy's case of diseased bladder - - - 463

